# Supplementary material for: Species-wide gene editing of a flowering regulator reveals hidden phenotypic variation
Source: PLoS Biol. 2025 Jun 9;23(6):e3003226. doi: 10.1371/journal.pbio.3003226 (PMC12185000; doi:10.1371/journal.pbio.3003226)
Supplement: Attachment — Submitted filename: Lutz_et_al_PLOS_Bio_Revision_Response_to_editors.pdf [file pbio.3003226.s024.pdf]

Changes made in main text during revision are marked in green

Comments and our responses:

Reviewer 1:

This study is an extensive, and extremely well-undertaken, analysis of FLC-independent variation in flowering time in A. thaliana. This work provides a large well characterized resource for the community, important for analysis of, for example, residual vernalization sensitivities in the absence of FLC, and flowering time loci responding to metabolic signals.

An important aspect of the paper is the elaboration of the broader (and generally underappreciated) pleiotropic roles in developmental and physiological processes of FLC within the A. thaliana species. The F2 populations derived from intercrosses of 20 flc knockout mutants identified a total of 115 additive QTL. These may well help the comparative analysis of flowering time variation in other species, where the effects of FLC are also not so strong. The effects of loci linked to FLC were also clearly revealed, which had been obscured in other analyses.

This work also reinforced the conclusion that another major factor in flowering time variation in A. thaliana is FT. Their analysis predicted that extremely early accessions should be found in nature - and they validated this by prospecting for early flowering plants in Southern Italian populations. The importance of major loci in flowering time variation was not what was expected by the evolutionary biologists so the conclusions from this analysis should be of broad interest.

Previous thinking had also suggested that inactivation of FLC led many accessions towards an early flowering/fast growth strategy. However, they found flc mutants that grew more slowly, opposite to what would be expected from the prevailing slow-fast-continuum. Again, follow ups using these new resources should provide important conclusions on evolutionary trade-offs.

Thank you!

Reviewer 2:

Lutz and colleagues have surveyed the multivariate phenotypic effects of FLC mutants, then explored the roles of secondary regulators within FLC mutant classes. This work predicted, then validated, that there are early flowering natural genotypes that are missed historically in collections. In addition to the direct empirical discoveries, the methods and experiments herein demonstrate the value of exploring residual variation when controlling for variation at "hub" genes; this approach could be applied to other systems and to aid the discovery of secondary loci that participate in biological processes but would otherwise be difficult to detect. Overall, I felt the text was clear and concise, the methods appropriate and well described, and the results exciting. This was a fun read! I have some comments and suggestions that I hope will improve future drafts.

Thank you!

Throughout, correlations are interpreted as causal and indicative of pleiotropy (etc.). This is only true if they are genetic correlations (taken from the breeding values of the genotypes) and not phenotypic correlations (taken across observations including replicates within genotype). Please include descriptions including sample size and indicate which type of correlation coefficient is being reported.

Please always include the sample size, test statistic and P-value when any difference (or correlation) is reported. For example, first paragraph of p4, and many other places throughout.

Good point. Throughout the text we have used the terms “correlation” and “correlated” more judiciously, to avoid giving the impression that all correlations are necessarily because of genetic causes (they clearly are not, as there is always more variance when plants flower later, simply because measurements become less precise and there is more time for stochastic environmental fluctuations to affect flowering). Furthermore, we have included details on statistics in all figure legends.

There were a couple places that I had trouble following the logic in the text. In general the text is very concise, so perhaps you could add a sentence or clause in these places to flesh out how causality can be inferred in these situations:
[1] "The relative acceleration of DTF in a mutant was positively correlated with DTF of the wild type, but much weaker for RLN and CLN, which suggests that FLC affects not only time to bolting, but also the leaf initiation rate (plastochron)" — its not clear why weaker correlations demonstrate that FLC differentially affects leaf initiation rate (couldn't it just be a less heritable phenotype?)

[2] "Also as expected, residual vernalization sensitivities (expressed as log2 ratio of flowering time without and with vernalization) were higher for the knockdown lines than the complete knockouts (Mann-Whitney U rank test, $p < 0.001$ for both DTF and RLN), highlighting how vernalization sensitivity can be tuned by modifying FLC expression and activity" (see comment above, couldn't non-genetic variance be driving this?)

Agreed. Indeed, non-genetic variance definitely contributes to differences in correlations. We have calculated broad-sense heritability (H^2) of flowering traits in the *flc* mutants and wild types and found that H^2 in the mutants overall was high but did not differ between traits. In the wild types RLN and CLN showed slightly lower H^2 than DTF and overall much lower H^2 than in the mutants. Thus, differences in heritability potentially contribute to some extent to differences between relative flowering acceleration in days and leaf formation. We have added more context in the text.

[3] "We conclude that FLC is a major component of trait de-canalization, or uncoupling" … "in contrast to the high correlations in the parental flc mutants (Fig. 1E), indicating that trait canalization due to FLC disruption". I think I get it, but it took me a while to figure out the logic about how FLC could both uncouple and couple traits.

The disruption of *flc* or vernalization only leads to different aspects of flowering being better coordinated. To avoid misunderstandings we have simplified the wording and use “coupling” and “uncoupling” to make it hopefully clearer.

The term "panspecific" is kind of jargon-y … I can understand that it means "all variation within a species", but the first hits for this term in a google search yielded results related to specificity of antibodies. I would strongly recommend using a different term or just describe what you mean.

We have removed the term “panspecific” and changed the title, taking a cue from a recent paper of the Lippman lab, which introduced the term “pan-genetic” (and which kindly made reference to our companion paper describing the generation of the *flc* mutant collection).

I had a bit of trouble following the flow of information in the multi-panel figures. Some things that could be cleared up:
[1] The RNA-seq analysis, shown in Figure 5, is limited to seven early flc mutants, but the remainder of the analyses shown in Figure 5 (panels C-E expand) to 29 flc mutants. Why were these additional genotypes included and are they also representative of early flowering flc mutants?

For RNA-seq, we chose a subset of early flowering mutants that represents most of the flowering spectrum of the *flc* lines. We have added more information about the selected lines to the text. We have also added more information about the number of lines covered in the different experiments (Fig. 5C-E)

[2] Figure 3A, Do the labeled arrows refer to the parents of the F2 population? If so, please indicate why not all parents used in crosses are labeled and what the significance is of the labeled genotypes.

The arrows indicate the earliest flowering parents of the five F_2 populations with a QTL at *FT*. We have added more information to the figure caption.

[3] The HSD labeling in Fig 3D is a bit distracting and doesn't add much. Are you really interested in all differences, and if so, are the groupings correcting for the many multiple tests (contrasts)?

We now use transparency for the labels to improve readability. And yes, the comparisons are corrected for multiple testing.

[4] Are the seven early flowering flc mutants (shown in Figure 5) represented by the Figure 3 cluster 1 earliest flowering mutants? I am missing the discussion that connects the differences in growth strategies to the FLC-independent loci that may be underlying differences in growth rates.

The seven lines used for RNA-seq belong to different clusters based on our growth analysis. Therefore, we do not have enough information to make statements about the possible contribution of the regulators unmasked by *FLC* as drivers of different growth strategies. We have added an explanation to the discussion in this regard:

“Since the early flowering *flc* lines that were examined by RNA-seq clustered in different groups in the growth analysis, they did not provide further insights into functions of *FLC*-independent regulators on pleiotropic phenotypes.”

[5] In the text, "the difference in AZI1 was not robust to correction for multiple comparison", however the relative expression levels of AZI1 of mutants compared to wild types is denoted as highly significant as shown in Figure 5C ($p \leq 0.01$; ***). See comment above about reporting of statistics.

Comparison of <u>all</u> mutants to <u>all</u> wild types revealed different *AZI1* levels. However, when we performed pairwise comparisons between <u>individual</u> mutants and the respective wild types, most differences did not remain significant after correction for multiple testing. We have added more details in the text for clarification.

[6] Figure 1C has dashed vertical lines that indicate 0 values … I think. Please describe in the caption.

Yes, the dashed line indicates 0. We added details in the figure caption.

[7] Figure 2D, its probably better to label each chromosome with the respective chromosome number (perhaps instead of the cM positions, since this is just scaled to the size of the facets).

We have labelled each chromosome now.

[8] Figure 3C, Is the location of the top panel image the "Angit site"?

Yes, it is the Angit site. We added this to the figure caption.

[9] Figure 4C, Assuming the solid, cluster specific horizontal lines are means (or medians?). Please describe in the caption.

Yes, the horizontal lines indicate the mean values. We added this information to the figure caption.

[10] Figure 5B, The color assigned to "response to oxygen-containing compound" exceeds the "Fold enrichment" color scheme range.

Indeed, the color scheme was limited to the top four groups. We have removed "response to oxygen-containing compound” as it anyways does not add much information.

There were some discrepancies between the SI figures and text:
[1] Figure S9 indicates that there may be haplotype-specific structural variation at the FT locus, but this figure or insight is not discussed in the main text. Also, for pane B of this supplemental figure, not all F2 populations are represented, which would be useful to establish the existing variation at the FT locus for all parents of F2 populations used in QTL

mapping. For the other six F2 families not represented in pane B, did the parents not have a QTL that colocalized in the FT genomic region?

Figure S9 was erroneously included in the manuscript, from a previous draft. We have removed this figure. The numbering of the following supplemental figures therefore changed: S10 → S9, S11 → S10, S12 → S11, S13 → S12.

[2] The text for Figures S10 and S11 is included in the file, but the figures are missing.

Indeed, Figures S10 and S11 were missing in the manuscript. Our apologies. They are included now – we hope they are informative.

[3] Figure S8, C) please indicate the green vertical line as positions of "a priori candidate genes" as denoted in the main text D) the blue and pink dots are referred to as "green" and "red".

We have added labels to Fig. S8.

Reviewer 3:

This paper focuses on the genetic basis of flowering time variation in Arabidopsis following mutation of one of the main flowering regulators, FLC. Genetic mapping when FLC is perturbed reveals new flowering loci and expands the range of flowering time diversity (and other phenotypes) in this species. This work is pretty neat and will likely be of interest to researchers in the Arabidopsis community and potentially beyond.

Thank you!

While I enjoyed reading this paper, there were several major concerns:

-Some terminology is used that is not common in genetics. What are panspecific mutants or prognostic sequence analysis? I would recommend avoiding such jargon and writing in simple language.

Good point. We have tried to reduce use of jargon throughout. Instead of panspecific we now use the term pan-genetic, which was recently introduced by the Lippman lab, which also kindly referred to our preprint describing generation of the *flc* mutant used in this paper.

-Understanding this manuscript, especially the panspecific mutants component, requires reading at least one prior paper. This current paper needs to be able to entirely stand on its own. A reader should not be expected to read another paper to be able to understand the current one. In the main text, the nature of the mutations and the accessions carrying them should be explicitly described. So much depends on that information and it is critical to make it simple to understand.

Good point. We have added more information about the new generation of *flc* mutant lines and the resource in general in the main text.

-More description is needed in the main text about the crosses. Which accessions/mutants are being crossed? Ideally there would be a figure or panel summarizing this in an easy-to-understand way. I found some of this information in the Supplement, but if asked, I could not fully explain the exact experimental design underlying this work.

We have added more information on the parental lines (*flc* knockout lines) used for crossing in the main text. Also, we have added a new supplemental figure S5B, indicating the parental pairs, their flowering times and the differences in flowering time.

-Connected to the above, the FLC region comes up as a locus in numerous crosses. If some of the mutants are just knockdowns, couldn't detection of the FLC locus simply reflect variation at FLC itself? This is a finding that connects back to the importance of clearly explaining the experimental design. If it is possible that FLC may itself be the QTG at the QTL, that would be good to present as an alternative explanation.

Only *flc* knockout lines were used as parental lines for the crosses. Therefore we should be able to exclude *FLC* as causal for the QTL on top of chromosome 5.

-The final section of the Results seemed tacked on. The prior section about the Italian strains from Angit and the detection of part of the phenotypic spectrum that was missed was compelling and a thoughtful extension of the preceding sections. However, the final Results section did not integrate as well with the other work. Maybe it can be better explained how the final presented results integrate with everything else. I did not think the final Results section was critical to the main thrust of the paper, but maybe I misunderstood it.

We indeed struggled a bit with this. We have tried to make the transitions between these sections more logical and to provide a fuller motivation for studying the broader pleiotropic roles of *FLC*. We agree that this part could be developed further into an independent story. Since the Weigel lab is, however, no longer recruiting new students and postdocs, and the first author has transitioned to industry, it is unlikely that this will happen and we therefore wanted to share the information in this context. If the reviewer feels that this is best relegated to the supplement, we would be happy to accommodate such a suggestion.

- Generally, the IDs used in figures are not easy to understand. There are many main text and supplemental panels containing these IDs. What accessions/mutants are being examined in each figure? Connected to this, often the labels are too small or cramped to easily read (e.g., Figs S3, S7, S9, S13).

We have added a description on the nomenclature of the *flc* lines at first appearance in the main text (caption of Fig. 3D). Also, we have increased the label sizes in the figures as much as possible.

-Figs S10 and S11 were absent from the document I could access. I downloaded multiple times, but they were absent every time, suggesting they might be missing from the submission.

Our apologies. We have added S10 and S11. Figure S9 was a leftover from a previous draft. The numbering of supplementary figures has changed as follows: S10 → S9, S11 → S10, S12 → S11, S13 → S12

There was also a minor typo: -In second sentence of introduction, delete 'it' in 'Flowering it is orchestrated….'

Thank you, fixed.
